# Supplementary material for: Additive manufacturing of strong silica sand structures enabled by polyethyleneimine binder
Source: Nat Commun. 2021 Aug 26;12:5144. doi: 10.1038/s41467-021-25463-0 (PMC8390701; doi:10.1038/s41467-021-25463-0)
Supplement: Supplementary file 1 — Supplementary Information [file 41467_2021_25463_MOESM1_ESM.pdf]

Supplementary Information for

Additive Manufacturing of Strong Silica Sand Structures enabled by Polyethyleneimine Binder

Dustin B. Gilmer [a, c], Lu Han [a], Michelle L. Lehmann [a, c], Derek H. Siddel [b], Guang Yang [a], Azhad U. Chowdhury [a], Benjamin L. Doughty [a], Amy M. Elliott [b*], Tomonori Saito [a*]

[a] Chemical Sciences Division, Oak Ridge National Laboratory, Oak Ridge, Tennessee 37831, United States
[b] Manufacturing Science Division, Oak Ridge National Laboratory, Oak Ridge, Tennessee 37831, United States
[c] The Bredesen Center for Interdisciplinary Research and Graduate Education, The University of Tennessee, Knoxville TN 37996, United States

This manuscript has been authored by UT-Battelle, LLC, under contract DE-AC05-00OR22725 with the US Department of Energy (DOE). The US government retains and the publisher, by accepting the article for publication, acknowledges that the US government retains a nonexclusive, paid-up, irrevocable, worldwide license to publish or reproduce the published form of this manuscript, or allow others to do so, for US government purposes. DOE will provide public access to these results of federally sponsored research in accordance with the DOE Public Access Plan (http://energy.gov/downloads/doe-public-access-plan).

Table of Contents

1. Supplementary Figures
2. Supplementary Tables
3. Supplementary Discussion

1. Supplementary Figures

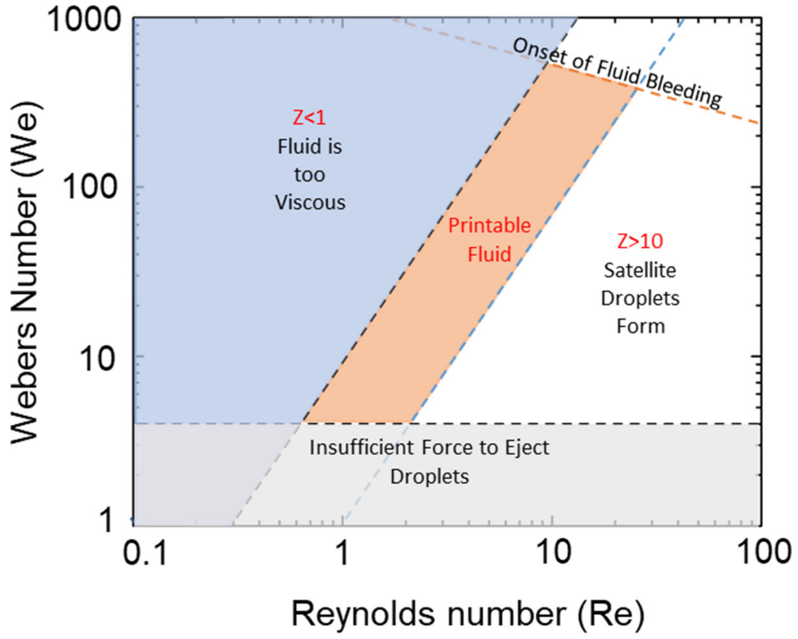

Supplementary Figure 1. Derby plot showing the printability range of inks using DOD print

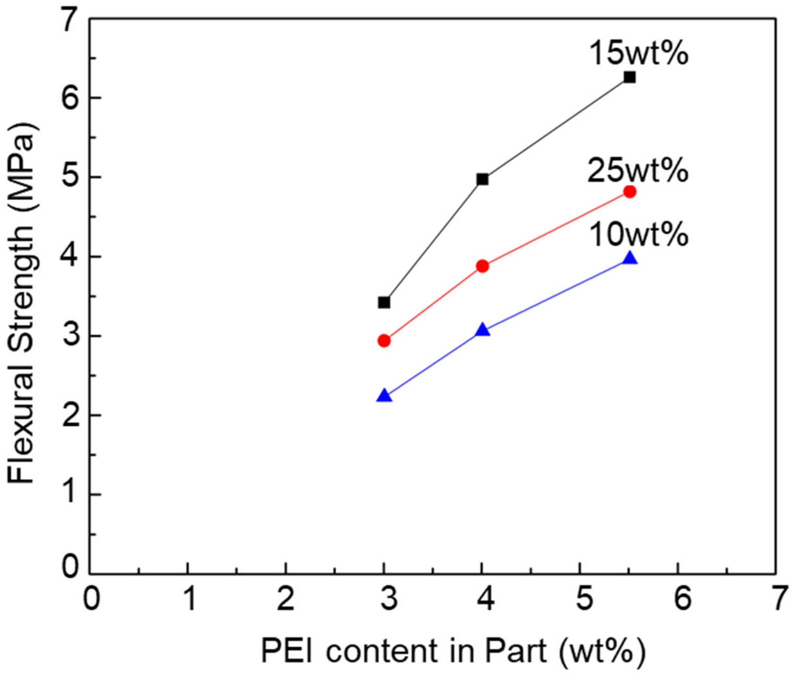

Supplementary Figure 2. Flexural strength comparison of printed parts with three solid loadings of PEI binder

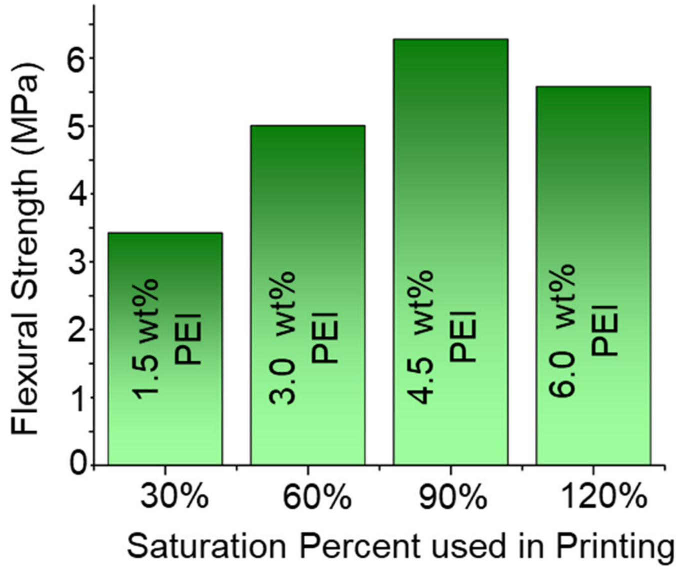

Supplementary Figure 3. Flexural strength of green parts printed with 15 wt% PEI binder at varying saturations, and correlation to PEI wt% in part

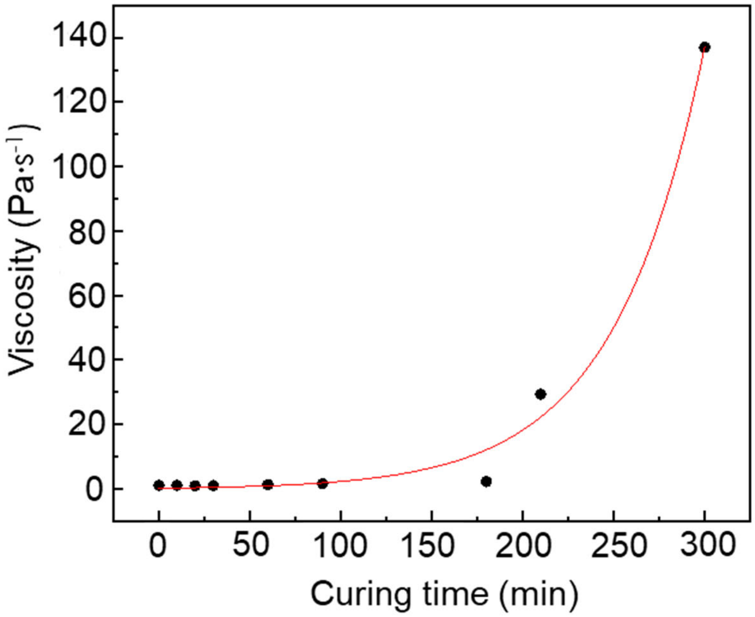

Supplementary Figure 4. Viscosity of different curing time of PEI with a polynomial fit.

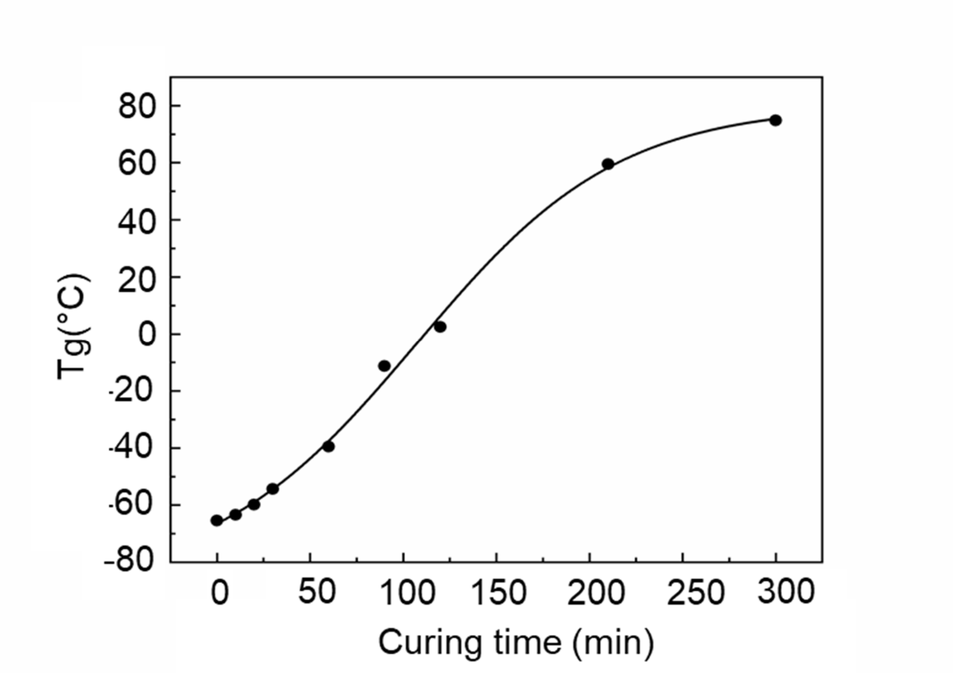

Supplementary Figure 5. The increase of T_g upon curing of PEI at 180°C.

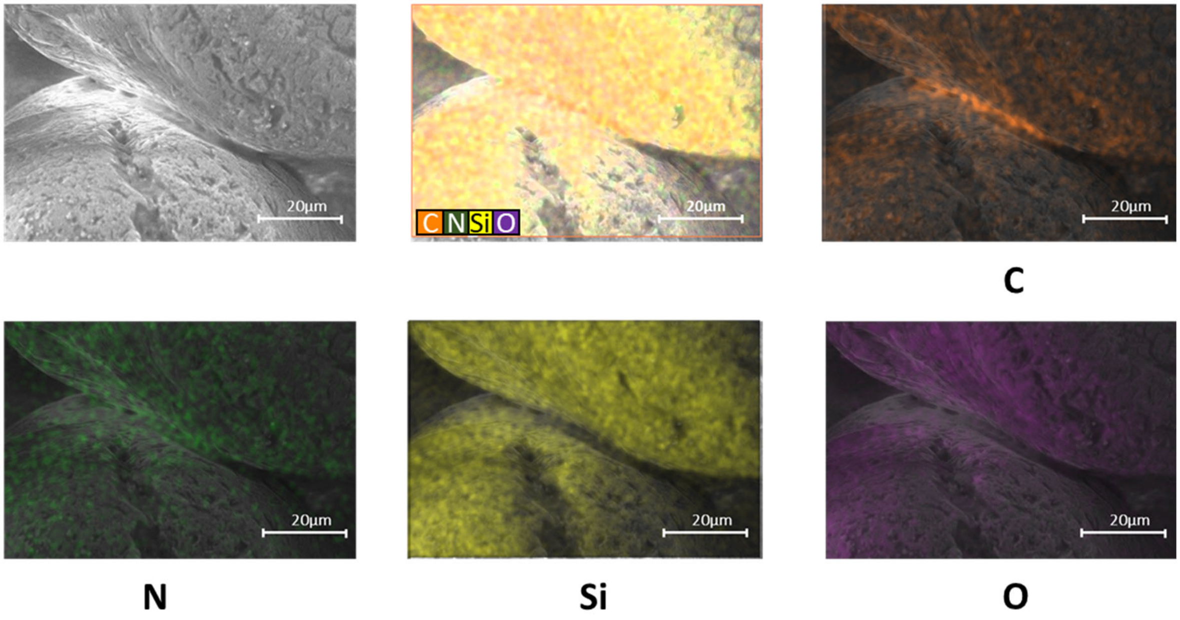

Supplementary Figure 6. SEM-EDX Images of printed PEI-silica sand, showing carbon, oxygen, silicon, and nitrogen

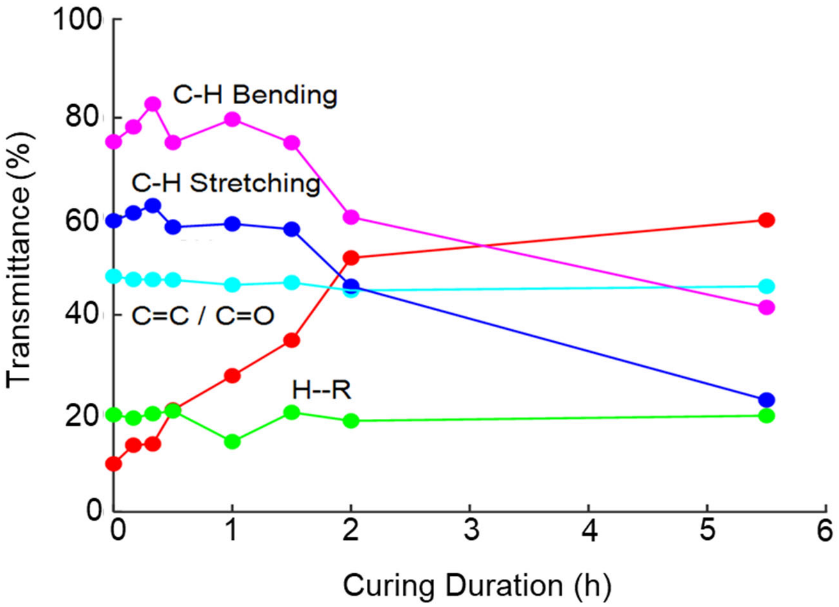

Supplementary Figure 7. FTIR spectra showing the increase in PEI oxidation

2. Supplementary Tables

Supplementary Table 1. Binder formulations with 75/25 H_2O/1-propanol solvent.

Polymer Loading (wt%)	Density (ρ) (g/mL)	Viscosity (mPa.s)	Surface Tension (mN/M)	Reynolds number (Re)	Webers Number (We)	Ohnesorge Number (Oh)	Z number
10	1.01	4.49	36.6	56.24	72.14	0.15	6.77
15	1.02	6.77	37.5	37.30	72.14	0.23	4.57
20	1.02	9.99	36.1	25.28	72.14	0.34	3.04
25	1.03	15.9	37.3	15.88	72.14	0.53	1.95

A nozzle radius (L) = 25 µm and a nozzle exit velocity (v) of 10 m/s are utilized in the calculation to determine if the fluid would form stable droplets utilizing DOD print modules with.

Supplementary Table 2. Effect of saturation on PEI content in part and flexural strength.

Printing Saturation	PEI content in part (wt%)	Flexural Strength (MPa)
30% Saturation	1.50	3.42
60% Saturation	3.00	5.00
90% Saturation	4.51	6.23
120% Saturation	6.01	5.58

Supplementary Table 3. Effects of temperatures on green strength on parts printed at 20% saturation.

Cure Temperature (°C)	Curing Time (h)	Strength (MPa)
180	2	2.94 ± 0.14
150	2	0.14 ± 0.02
125	2	Not measurable

Supplementary Table 4. CTE of silica sand samples printed with PEI and CTE of parts infiltrated with ECA

Material	CTE (µm/m°C) at 185 °C
Silica Sand Printed with PEI	14.73
Infiltrated with ECA	20.81
PolyECA	96.10

3. Supplementary Discussion

Droplet formation in drop-on-demand (DOD) printing is a complex process that requires specific viscosities and surface tensions which can be characterized by the Webers (Eq.1) and Reynolds numbers (Eq.2), where μ is dynamic viscosity, ρ is the density, γ is the surface tension of the binder, v is the velocity at which the droplet is ejected, and L is the characteristic length which equates to the nozzle radius. These parameters are combined to form the Ohnesorge number (Oh) (Eq.3), a parameter utilized to find the Z number (Z =1/Oh), a dimensionless constant that illustrates printable fluids based off fluid properties[1,2]. It has been determined that a Z range from 10 > Z > 1 will form stable droplets and the printable range is represented in (Supplementary Fig. 1)[1].

$$We = \frac{\rho v^2 L}{\gamma} \quad (1)$$

$$Re = \frac{\rho v L}{u} \quad (2)$$

$$Oh = \frac{\sqrt{We}}{Re} = \frac{u}{\sqrt{\gamma \rho L}} \quad (3)$$

PEI can be dispersed into a solvent mixture of 75 wt% H_2O and 25wt% 1-propanol to tune its viscosity and surface tension for printability. PEI in 75/25 H_2O/1-propanol provides an array of binder formulations, which meets fluid property requirements for printing with varying solid loadings of PEI polymer (Supplementary Table.1). The binder formulations were each tested by evaluating flexural strength of printed parts with varied PEI content. A 15wt% solid loading was found to provide the optimum strength when utilizing the silica sand powder compared to other binder formulation (Supplementary Fig. 2).

In addition to optimizing the binder formulation with a variety of PEI loadings in the ink, the optimal binder saturation in the printing process was evaluated to produce parts with the maximal green strength. To achieve the maximum green strength, the binder saturation was systematically increased, which is proportional to the increased wt% of the polymer in the part. The flexural strength as a function of saturation with correlation to the PEI wt% in the part is described in Supplementary Fig. 3 and Supplementary Table 2.

Curing after printing plays a critical role in the final strength of the green part. When testing the green strength at different curing temperatures, any curing temperature below 180°C imparted lower strength (Supplementary Table 3). To study how heat altered the physical properties of PEI upon curing, PEI was cured at 180 °C for different times to study the curing mechanism. The color of PEI became darker under curing, indicating the occurrence of oxidation, which was confirmed utilizing spectroscopic characterization methods in the main text. With the increase of curing time, the viscosity of the binder also decreases slightly in the first 20 minutes and increase dramatically after 1.5 hours due to the oxidation process (Supplementary Fig. 4).

Differential Scanning Calorimetry (DSC) was used to measure glass transition temperature (T_g) of PEI and cured PEI using modulated DSC (TA Instruments Q1000). The heating procedure was set to modulate ±1.00 °C every 60 s at the rate of 3°C/min from -160 to 90°C. It can be clearly observed that the T_g increases with increased curing time (Supplementary Fig. 5). With the increase of the curing time, the increased oxidation and subsequent cross-linking results in more intramolecular and intermolecular interactions, leading to higher T_g values.

The increased T_g caused by longer curing times (Supplementary Fig. 5) along with the enhanced interfacial interactions indicates that the thermal curing of PEI is integral in achieving high strengths due to the

chemical and conformational change. It is important to note that as the size of parts are increased through the printing on larger systems, the duration of the thermal curing cycle should be increased to account for delay in reaching the curing temperature in the part. For example, significantly large parts may need to be cured for overnight (e.g., 18h) to make sure the chemical and conformational changes taking place uniformly throughout the part.

Scanning electron microscope (Hitachi TM3030) and energy-dispersive X-ray spectroscopy (SEM-EDX) was utilized to visualize the binding modalities and chemical species distribution (Supplementary Fig. 6). Due to the capillary effect, the binder concentrates between adjacent particles and forms a neck. Carbon and nitrogen from the PEI can be observed at the necks of the silica/polymer interface and the presence of silicon and oxygen on the silica particles.

The FTIR spectra in (Supplementary Fig. 7) shows the change in transmittance of the FTIR spectra given in the main text. It clearly illustrates the oxidations of the PEI species with the increase in transmittances of the C=C and C=O and C-H stretching, caused by curing the polymer at 180°C.

In Sum Frequency Generation (SFG) measurements, two laser pulses were focused onto the sample: one is centered in the mid-infrared (IR) spectral region and the other one in the near infrared (NIR). The simultaneous interaction of these two intense laser fields drives a second order polarization in the sample that oscillates at the sum (and difference) frequency of the incident optical fields and therefore generates new colors of light. The radiated light is descriptive of the interfacial layer where bulk symmetry is broken[3–5]. The radiated SFG intensity is proportional to the absolute square of the effective second order nonlinear susceptibility, $\chi_{\text{eff}}^{(2)}$, and the driving laser fields, (E_{IR} and E_{NIR}):

$$I_{SFG} \propto \left|\chi^{(2)}_{\text{eff}} E_{IR} E_{NIR}\right|^2 \qquad (1)$$

The effective nonlinear susceptibility contains a resonant ($\chi^{(2)}_{\text{res}}$) and non-resonant ($\chi^{(2)}_{\text{NR}}$) contribution as given by:

$$\chi^{(2)}_{\text{eff}} = \chi^{(2)}_{\text{NR}} + \chi^{(2)}_{\text{res}} = \chi^{(2)}_{\text{NR}} + \sum_q \frac{A_q}{\omega_{IR} - \omega_q + i\Gamma_q} \qquad (2)$$

where ω_{IR} is the frequency of incident IR laser light, A_q is an amplitude, ω_q is the resonant frequency, and Γ_q is related to the linewidth of the qth vibrational mode. Eq. 2 shows that the SFG signal is enhanced when a vibrational frequency matches a component in the broadband IR pulse. The sensitivity to the interfacial layer originates from the even-order field interaction, which based on symmetry arguments, is forbidden in centrosymmetric and isotropic bulk media[3–5]. A such, the SFG spectrum is analogous to conventional linear vibrational spectroscopies (Raman or IR absorption) but selectively reports on the interfacial layer. Therefore, SFG can be used to understand the interfacial interactions taking place on the molecular level between quartz and PEI.

The porosity of the printed and infiltrated samples were analyzed by a Zeiss Metrotom 800 X-Ray CT (XRT) system. This XRT system reports the density variations within samples, that can differentiate between areas of high and low density or in the areas where voids are present (Supplementary Video 1). This can then be used to describe the porosity of a given sample in bulk terms. XRT determined that the green samples had a porosity of 9.90 % and 8.92 % after ECA infiltration, indicating that ~1% of the total porosity was filled in with the ECA in-situ polymerization.

A thermomechanical analysis was performed on the printed and infiltrated materials to elucidate the Coefficient of Thermal Expansion (CTE), and the results are shown in Supplementary Table 4. Here, the plain printed silica sand with PEI yielded an average CTE of 14.73 µm/m°C at 185°C. Once infiltrated with ECA, the CTE increased to 20.81 µm/m°C at 185°C, which is expected from higher CTE of polyECA,

96.10 μm/m°C at 185°C. These low CTE values makes them well suited for uses in tooling applications such as composite layups.

Supplementary References

1. Derby, B. Inkjet Printing of Functional and Structural Materials: Fluid Property Requirements, Feature Stability, and Resolution. *Annu. Rev. Mater. Res.* 40, 395–414 (2010).
2. Van Hoeve, W. *et al. Breakup of diminutive Rayleigh jets*. (2010).
3. Shen, Y. R. Surface properties probed by second-harmonic and sum-frequency generation. *Nature* 337, 519–525 (1989).
4. Lambert, A. G., Davies, P. B. & Neivandt, D. J. Implementing the Theory of Sum Frequency Generation Vibrational Spectroscopy: A Tutorial Review. *Appl. Spectrosc. Rev.* 40, 103–145 (2005).
5. Wang, H. F., Gan, W., Lu, R., Rao, Y. & Wu, B. H. Quantitative spectral and orientational analysis in surface sum frequency generation vibrational spectroscopy (SFG-VS). *International Reviews in Physical Chemistry* 24, 191–256 (2005).
