# Supplementary material for: Additive manufacturing of strong silica sand structures enabled by polyethyleneimine binder
Source: Nat Commun. 2021 Aug 26;12:5144. doi: 10.1038/s41467-021-25463-0 (PMC8390701; doi:10.1038/s41467-021-25463-0)
Supplement: Supplementary file 3 — Description of Additional Supplementary Files [file 41467_2021_25463_MOESM3_ESM.pdf]

Description of Additional Supplementary Files

File Name: Supplementary Movie 1
Description: XRT of Printed Sand with PEI Binder
